# Alternative methods of interpreting quality of life data in advanced gastrointestinal cancer patients

**DOI:** 10.1054/bjoc.2001.2046

**Published:** 2001-09-01

**Authors:** K Nordin, J Steel, K Hoffman, B Glimelius

**Affiliations:** Department of Public Health and Caring Sciences, Uppsala University, Uppsala, Sweden; Department of Oncology, Radiology and Clinical Immunology, Akademiska Sjukhuset, Uppsala, Sweden

**Keywords:** quality of life, advanced gastrointestinal cancer, palliation, EORTC QLQ-C30

## Abstract

Understanding of how to analyse and interpret quality of life (QoL) data from clinical trials in patients with advanced cancer is limited. In order to increase the knowledge about the possibilities of drawing conclusions from QoL data of these patients, data from 2 trials were reanalysed. A total of 113 patients with pancreatic, biliary or gastric cancer were included in 2 randomised trials comparing chemotherapy and best supportive care (BSC) with BSC alone. Patient benefit was evaluated by the treating physician (subjective response) and by using selected scales and different summary measures of the EORTC QLQ-C30 questionnaire. An increasing number of drop-outs (mainly due to death) with time did not occur in a random fashion. Therefore, the mean scores in the different subscales of the QLQ-C30 obtained during the follow-up of interviewed patients did not reflect the outcome of the randomised population. The scores of the patient-provided summary measure, ‘Global health status/QoL’, were stable in a rather high proportion of the patients and could not discriminate between the 2 groups. 3 other summary measures revealed greater variability, and they all discriminated between the 2 groups. A high agreement was also seen between the changes in the summary measures and the subjective response. A categorisation of whether an individual patient had benefited or not from the intervention could overcome the problem with the selective attrition. © 2001 Cancer Research Campaign


*British Journal of Cancer* (2001) **85**(9), 1265–1272
© 2001 Cancer Research Campaign
doi: 10.1054/ bjoc.2001.2046, available online at http://www.idealibrary.com on IDEAL

http://www.bjcancer.com

### Alternative methods of interpreting quality of life data in advanced gastrointestinal cancer patients

**K Nordin[1], J Steel[2], K Hoffman[2] and B Glimelius[2]**

[1]Department of Public Health and Caring Sciences, Uppsala University, Uppsala, Sweden; [2]Department of Oncology, Radiology, and Clinical Immunology, Akademiska Sjukhuset, Uppsala, Sweden

**Summary** Understanding of how to analyse and interpret quality of life (QoL) data from clinical trials in patients with advanced cancer is limited. In order to increase the knowledge about the possibilities of drawing conclusions from QoL data of these patients, data from 2 trials were reanalysed. A total of 113 patients with pancreatic, biliary or gastric cancer were included in 2 randomised trials comparing chemotherapy and best supportive care (BSC) with BSC alone. Patient benefit was evaluated by the treating physician (subjective response) and by using selected scales and different summary measures of the EORTC QLQ-C30 questionnaire. An increasing number of drop-outs (mainly due to death) with time did not occur in a random fashion. Therefore, the mean scores in the different subscales of the QLQ-C30 obtained during the follow-up of interviewed patients did not reflect the outcome of the randomised population. The scores of the patient-provided summary measure, 'Global health status/QoL', were stable in a rather high proportion of the patients and could not discriminate between the 2 groups. 3 other summary measures revealed greater variability, and they all discriminated between the 2 groups. A high agreement was also seen between the changes in the summary measures and the subjective response. A categorisation of whether an individual patient had benefited or not from the intervention could overcome the problem with the selective attrition. © 2001 Cancer Research Campaign

**Keywords**: quality of life; advanced gastrointestinal cancer; palliation; EORTC QLQ-C30

It has been increasingly recognised that recordings of the influence on the patients general well-being or quality of life (QoL) are important in randomised clinical trials designed to test the efficacy of new treatments for cancer. The traditional physician-defined endpoints such as survival, tumour response and toxicity have been supplemented with patient-defined end-points, using instruments such as the European Organisation of Research and Treatment of Cancer Quality of Life Questionnaire (EORTC, QLQ-C30) (Aaronson et al, 1993) and several others (de Haes et al, 1990; Schag et al, 1991; Cella et al, 1993). However, the use of QoL assessments in clinical trials has been rather limited, although increasing from 1.5 to 8.2% during 1980–1997 (Sanders et al, 1998). QoL assessments are also in need of further methodological improvement before this endpoint can be regarded as fully established with respect to its ability to provide unequivocally useful data in clinical trials (Gunnars et al, 2001).

QoL assessments are important not only in randomised clinical trials, but also in the everyday clinical practice. For most patients with advanced cancer, the primary goal of therapy is palliation, and therefore it is particularly important to consider QoL factors in the evaluation of these treatments (Guyatt et al, 1989). However, Detmar et al (2000) found that only in a minority of cases did QoL aspects play a significant role in the everyday clinical practice in decisions of palliative chemotherapy. When asking oncologists, the barriers to use QoL as a method of monitoring the response to palliative treatment were time and resource constraints and perceived lack of appropriate, clinically sound instruments (Morris et al, 1998).

*Received 2 March 2001*
*Revised 22 June 2001*
*Accepted 13 July 2001*

*Correspondence to:* K Nordin

Important reasons for difficulties in assessing QoL in clinical trials involving patients with advanced cancer includes attrition secondary to patient illness or death due to underlying malignancy; ceiling effects of items and scales; content validity of scales; and the possibility that our current methods are not sophisticated enough to capture changes. The problems with attrition also entail that the data will become more biased at each follow-up because only survivors who have better QoL than the patients dying or already dead are included. Attrition due to death or too poor performance seems to be unavoidable, but strategies to minimise the problem have been suggested (Bernhard et al, 1998). Also, statistical methods to handle data that are missing not at random are described, but are more complex compared to methods for handling data missing at random (Troxel et al, 1998). Understanding of how to effectively analyse and interpret data from trials in patients with advanced cancer and present the data in a relevant fashion in order to guide researchers and clinicians remain limited. A categorisation of whether a patient has benefited from treatment, at least to a certain extent, in analogy with whether an objective tumour regression (response) has occurred or not, would facilitate understanding of QoL data from clinical trials.

In 1993, Lydick and Epstein (1993) suggested an alternative to traditional statistical methods of interpreting QoL data. They recommended the use of an anchor-based interpretation of data derived by QoL instruments by comparing changes in QoL data to changes in other ratings or clinical changes. In addition, Sprangers and colleagues (1999a) have recently recommended that researchers who do not detect differences using core or disease-specific QoL

instruments identify, a priori, a limited set of questionnaire subscales and single items considered to be of primary interest to use as summary scores.

In a series of trials recruiting patients with advanced symptomatic gastrointestinal cancers, who untreated have median survivals of 2–5 months, we have explored the value of palliative chemotherapy. The traditional, physician-defined endpoints, such as survival and objective and subjective response rates, have been supplemented with patient-defined endpoints. In the first trials, mainly comprising patients with colorectal cancer, we used an Uppsala questionnaire designed in 1985 (Glimelius et al, 1989, 1992, 1994), but have since 1992 gone over to the EORTC QLQ C-30 (Aaronson et al, 1993) since this is an internationally tested questionnaire. The difficulties with compliance, attrition, complexity of the data and trade-off are, however, identical irrespective of which instrument is used. In order to increase our knowledge about the possibilities of drawing conclusions from QoL data of the patients, we have reanalysed the data from 2 randomised trials, one recruiting patients with pancreatic-biliary cancer (Glimelius et al, 1996) and one patient suffering from gastric cancer (Glimelius et al, 1997). In both trials, chemotherapy and best supportive care (BSC) were compared with BSC alone. The aims of the present study are 2-fold: (1) to explore meaningful alternatives of analysing and interpreting QoL data to facilitate detection of changes in QoL between treatment groups in patients with advanced gastrointestinal cancer, and (2) to compare these alternative methods of analysing and interpreting QoL data.

### PATIENTS AND METHODS

### Patients

Between June 1992 and February 1995, a total of 120 patients were recruited from 6 hospitals in Sweden for 2 parallel, multicentre randomised trials. The patients were either randomised to immediate chemotherapy including BSC or to BSC only, with a possibility for chemotherapy if BSC did not result in palliation. At randomisation, 113 patients (94%) (44 pancreatic cancer, 26 biliary cancer, and 43 gastric cancer patients) completed the EORTC QLQ-C30. Inclusion and exclusion criteria have been described elsewhere (Glimelius et al, 1996, 1997). Selected patient characteristics are presented in Table 1.

### Treatment

For patients randomised to the chemotherapy group, the chemotherapy regimen was sequential 5-fluorouracil and leucovorin, either alone (Nordic Gastrointestinal Tumor Adjuvant Therapy Group, 1993) in patients above the age of 60 years with a Karnofsky performance status (KPS) of 70 or less or combined with etoposide. In gastric cancer patients, the ELF regimen (Wilke et al, 1991) was used whereas in pancreatic or biliary cancer, a modification of ELF, vizually FELv, was used (Glimelius et al, 1996).

### Evaluation of treatment effects

The evaluation of treatment effects followed a series of predetermined analyses based upon prospective data recordings. These included subjective response evaluations made by the treating physician, and QoL evaluations using the EORTC QLQ-C30 version 1.0 (Aaronson et al, 1993; Fayers et al, 1995). The EORTC QLQ-C30 was completed by the patients immediately prior to randomisation and after 2 and 4 months prior to the tumour evaluations. All scales were linearly transformed to a 0–100 scale. High scores on the functional scales refer to a better level of functioning while a higher score on the symptoms scale and single items means that the patient is experiencing a higher degree of symptomatology. Missing values were handled as recommended by the EORTC manual (Fayers et al, 1995), however, missing data were infrequent as 80% or more of the items were completed by all patients over the course of the study.

The data from the EORTC QLQ-C30 were presented using the traditional average global and scale scores (0–100) and four alternative methods.

**(1) A QoL rating** was performed by 2 independent raters of the average scale scores of the QLQ-C30. The raters were blind to group assignment and all clinical information. The patients were rated as 'improved', 'unchanged' or 'worse' compared to the previous time. The criteria (Glimelius et al, 1996) were

**Table 1** Patient characteristics at randomisation

| | Chemotherapy | Best supportive care |
|---|---|---|
| *n* | 57 | 56 |
| Tumour site | | |
| Pancreatic | 24 | 20 |
| Biliary | 12 | 14 |
| Gastric | 22 | 21 |
| Age, median, (range) | 64 (43–75) | 64 (40–75) |
| KPS, median (range) | 80 (100–50) | 80 (100–50) |
| Type of symptoms | | |
| Pain | 32 | 33 |
| Weight loss | 31 | 23 |
| Tiredness | 25 | 16 |
| Nausea/vomiting | 10 | 9 |
| Trouble swallowing | 7 | 7 |
| Others | 18 | 11 |
| Type of chemotherapy | | |
| ELF | 13 | 2 |
| FELv | 27 | 0 |
| FLv | 16 | 8 |

*© 2001 Cancer Research Campaign*

simple and agreement was high between the 2 raters (90%). In the case of discrepancies, a consensus was reached through discussions by the 2 raters.

**(2) Changes in global health status/QoL** (composed by the 2 questions in the QLQ-C30) was based on at least half a standard deviation movement from baseline, as recommended to be of clinical relevance (Lydick and Epstein, 1993; Juniper et al, 1994; Osboa et al, 1998). Depending upon the size and direction of any change, a patient could receive either a rating of 'favourable', 'unfavourable' or 'unchanged' at each time interval. Patients from the upper and lower 15% of the sample were scored as favourable or unfavourable, respectively, if they continued to score in the upper or lower 15% at subsequent time intervals, even if their scores did not change by more than half a standard deviation.

**(3) A summary score** based on the sum of all items in the scale, except the question regarding financial problems due to the limited variance and low score of this item secondary to Sweden's health care system. From the sum of the 5 functional scales and global health status/QoL score, where 100 is the best score, the sum of the 3 symptom scales and 5 of the single items, where 0 is the best score, was subtracted. The summary score could range from a minimum of –800 to a maximum of +600. In analogy with the categorisation performed for the global health status/QoL, described above, a favourable outcome was present if the scale scores remained above 450 (15% of patients had this score at baseline), or improved by at least 100 points (1/2 SD was 102). If the summary score at baseline was negative (15% had this at baseline, minimum –289), the score had to be positive at the follow-up evaluation in order to be classified as improved. Unchanged summary scores were those scores that remained within +/– 100 points and a worsening if the score decreased by more than 100 points or remained negative.

**(4) A limited QoL categorisation** was based on the physical functioning, emotional functioning, global health status/QoL scales, and the sum of the symptoms scales which included fatigue, nausea/vomiting, pain and the single items appetite and diarrhoea. These scales were considered to be of greater relevance than the others in advanced gastrointestinal cancer treated with 5FU-based chemotherapy. To select particular symptoms and single QoL items of primary interest in the evaluation of QoL has been recommended (Bernhard et al, 1999; Sprangers et al, 1999a). The categorisation followed principles used in the Clinical Benefit Response evaluation of pancreatic cancer patients suggested by Rothenburg et al (1996). A favourable outcome was present if the patient improved in at least one of the domains (physical function by at least 20 points, emotional functioning and global health status/QoL by at least 17 points), and the sum of the symptoms had decreased at least 50% or at least 50 points, if the sum was initially below 100, without any negative change in any other domain. A negative change was present in physical functioning if the scale decreased by more than 20 points or to a score of 20 points. Emotional functioning and global health status/QoL was considered to be significantly deteriorated if it decreased by 17 points or more and the sum of the symptoms scales by 50 points or more.

All patients who did not complete the QLQ-C30 at 2 and 4 months were considered to have an unfavourable response on methods 1–4.

Physician subjective ratings were recorded to provide subjective information to contrast with the others methods of interpreting the QLQ-C30 data. Based on personal interviews conducted at each follow-up visit, which occurred every second or third week, the treating physician gathered information from each patient concerning: (a) presence of signs or symptoms secondary to the tumour, (b) presence of symptoms associated with treatment toxicity and (c) KPS. The physician rated each patient, based on the above criteria as either 'symptom-free', 'improved', 'unchanged' or 'deteriorated'. The rating of 'symptom-free or improved' was given if the patient reported no or decreased tumour-related symptoms, in the absence of any of the WHO subjective grade III or IV treatment toxicities.

### Statistical analyses

The evaluation of treatment effects followed a series of predetermined analyses based on prospective data recordings. All analyses were performed according to the 'intention-to-treat' principle. Differences between proportions were tested using $\chi^2$ analyses and differences between means were tested using a 2-tailed Student *t*-test. Significance testing of changes over time on the subgroups of patients assessed at all time points was performed using 2-tailed repeated measures analyses of variance (ANOVA). Cohen's kappa was used to calculate agreement between categorisations based on the patient QoL scores and the physicians' subjective ratings. Survival differences were tested using the log-rank test. All statistical analyses were performed using Statistica 4.1.

### RESULTS

No significant differences in demographic information were found between the chemotherapy and the BSC groups at randomisation. The chemotherapy group had a median survival of 6 months whereas the BSC group had a median survival of 3 months (log rank test, $P < 0.01$).

The BSC group had significantly greater attrition when compared to the chemotherapy group (Table 2). After 2 months, 83% (45/54) of those interviewed at baseline completed the QLQ-C30 in the chemotherapy group and 61% (32/52) in the BSC treatment group. At 4 months, 50% (27/54) and 29% (15/52) completed the QLQ-C30 in the chemotherapy and BSC treatment group, respectively. In all but one patient, the reason for not completing the QLQ-C30 at 2 and 4 months was secondary to progressive disease with deterioration and inevitable death. Still living patients (2 in each group at 2 months and 5 in each group at 4 months) who did not complete the QLQ-C30 at the scheduled time had a range of survival of 2 to 38 days, median 8 days, after scheduled assessment, and KPS of 70 to 20, median 50. Patients who did not complete a questionnaire at 2 and 4 months were considered to have an 'unfavourable' outcome using each of the methods of analysing QoL. The physicians classified all these patients as 'worsened'.

### Use of the QLQ-C30 traditional average scale scores

No significant differences between treatment groups were found using the 5 functional scales, the global score, or the symptom scales/items at randomisation, at 2 or 4 months (Table 2). In the patients interviewed, all average scale scores remained at approximately the same level during the 4 months. However, when the

© 2001 Cancer Research Campaign 

**Table 2** Quality of life scores (Sd) at randomisation (R) and at T1 (2 months) and T2 (4 months) in the chemotherapy and best supportive care groups

| QLQ-C30 Scales/Items | Chemotherapy | | | Best supportive care | | |
|---|---|---|---|---|---|---|
| | R | T1 | T2 | R | T1 | T2 |
| Number of patients | 54 | 45 | 27 | 52 | 32 | 15 |
| Functional scales | | | | | | |
| Physical | 74 (28) | 72 (29) | 69 (27) | 74 (27) | 67 (31) | 73 (25) |
| Role | 56 (35) | 66 (40) | 58 (41) | 70 (32) | 63 (34) | 72 (37) |
| Emotional | 65 (22) | 75 (21) | 75 (21) | 68 (23) | 65 (23) | 66 (25) |
| Cognitive | 84 (18) | 84 (21) | 81 (20) | 84 (20) | 83 (21) | 76 (26) |
| Social | 75 (30) | 77 (29) | 76 (31) | 74 (29) | 73 (32) | 75 (30) |
| Global health status | 55 (18) | 59 (23) | 61 (20) | 58 (22) | 59 (26) | 56 (22) |
| Symptom scales/items | | | | | | |
| Pain | 32 (28) | 20 (26) | 21 (31) | 24 (27) | 26 (28) | 25 (28) |
| Fatigue | 45 (30) | 44 (27) | 45 (29) | 40 (30) | 46 (33) | 44 (25) |
| Nausea/vomiting | 21 (24) | 19 (24) | 23 (32) | 15 (24) | 24 (34) | 22 (19) |
| Dyspnoea | 17 (27) | 18 (22) | 18 (27) | 21 (25) | 28 (31) | 30 (26) |
| Insomnia | 30 (36) | 18 (29) | 14 (21) | 32 (38) | 27 (29) | 27 (33) |
| Constipation | 23 (31) | 14 (20) | 19 (28) | 22 (31) | 31 (35) | 19 (28) |
| Diarrhoea | 15 (26) | 18 (30) | 22 (32) | 11 (21) | 17 (26) | 20 (21) |
| Appetite | 41 (37) | 30 (34) | 29 (36) | 38 (37) | 40 (39) | 33 (36) |
| Financial | 4 (13) | 7 (17) | 8 (24) | 5 (16) | 7 (17) | 20 (30) |
| Summary score | 207 (206) | 254 (231) | 222 (237) | 236 (203) | 215 (256) | 268 (221) |

**Table 3** Number (and proportions)[a] of patients with favourable quality of life response using different methods of evaluating quality of life after 2 and 4 months of treatment

| Evaluation method | Chemotherapy | | Best supportive care | |
|---|---|---|---|---|
| | 2 months | 4 months | 2 months | 4 months |
| Global health status | 16 (29) | 13 (24) | 10 (20) | 5 (10) |
| QoL rating | 24 (44**) | 18 (33*) | 10 (20) | 6 (12) |
| Summary score | 21 (38*) | 14 (25*) | 7 (14) | 5 (10) |
| Limited QoL | 27 (49*) | 18 (33*) | 13 (25) | 6 (12) |
| Physician rating | 28 (52**) | 21 (39*) | 11 (21) | 9 (17) |

[a] The figure within the parentheses refers to the percentage of responders based upon the number of patients answering the QLQ-C30 at baseline. Statistical significance between the chemotherapy group and BSC group is shown by *$P < 0.05$ and **$P < 0.01$.

analyses were restricted to the subgroups of patients interviewed 2 or 3 times, a statistically significant decrease ($P < 0.05$) on the physical functioning scale and a statistically significant increase ($P < 0.05$) on the fatigue scale were seen for the BSC group (data not shown).

### Alternative methods of analysing the QLQ-C30

Initial analyses demonstrated that no significant differences were detected between the 2 randomisation groups when using the global score of the QLQ-C30. However, when using the alternative methods of analysing QoL data, based on all patients who answered the QLQ-C30 at randomisation, significantly higher proportions of patients who were randomised to the chemotherapy group had a favourable QoL when compared to the patients who received BSC (Table 3). If estimations were based only on those patients who answered the questionnaire after 2 or 4 months, differences were statistically significant, between groups, for only the QoL rating method (data not shown).

Using Cohen's kappa, a relatively high level of agreement was found between the QoL rating and summary score (Cohen's kappa = 0.68; Table 4). However, the agreement between the global health status/QoL score and summary scores was less robust with a Cohen's kappa coefficient of 0.45.

### Physician subjective ratings and agreements with the alternative methods of analysing the QLQ-C30

In the chemotherapy group, the treating physician considered 28 (52%) of the 54 patients answering the QLQ-C30 at randomisation to have had either a continuously symptom-free period or improved symptomatology in the absence of severe toxicity 2 months after randomisation (Table 3). The corresponding figure after 4 months was 21 (39%) patients. These numbers were significantly lower in the BSC group.

When the agreements between a subjective response and the QoL assessments were tested, concordance was seen in most patients using the QoL ratings (Cohen's kappa = 0.82) where only 7 (9%) patients were categorised differently (Table 5). The agreement between a subjective response and the limited QoL categorisation was also high (Cohen's kappa = 0.79) whereas it was moderate with the summary scores (Cohen's kappa = 0.53) and lower with global health status/QoL (Cohen's kappa = 0.35). The above pattern of results was similar at 4 months (data not presented). The great number of discrepancies (25, 33%) seen between a subjective response and changes in global health status/QoL was mainly due to the fact that the latter scores often remained unchanged.

 *© 2001 Cancer Research Campaign*

**Table 4** Agreement between the QoL rating, global health status/QoL and summary scores

| **QoL rating** | **Summary score** | | | |
|---|---|---|---|---|
| | **Improved** | **Unchanged** | **Worse** | **Total** |
| Improved | 28 | 6 | 0 | **34** |
| Unchanged | 4 | 12 | 5 | **21** |
| Worse | 1 | 0 | 21 | **22** |
| **Total** | **33** | **18** | **26** | **77** |
| Cohen's kappa = 0.68 | | | | |

| **Global health status/QoL** | **Summary score** | | | |
|---|---|---|---|---|
| | **Improved** | **Unchanged** | **Worse** | **Total** |
| Improved | 23 | 5 | 2 | **30** |
| Unchanged | 10 | 11 | 10 | **31** |
| Worse | 0 | 1 | 15 | **16** |
| **Total** | **33** | **18** | **26** | **77** |
| Cohen's kappa = 0.45 | | | | |

**Table 5** Agreement between the physician subjective ratings and quality of life evaluations

| | **QoL rating** | | **Global health status** | | **Summary score** | | **Limited QoL** | |
|---|---|---|---|---|---|---|---|---|
| | **Favour** | **Unfav** | **Favour** | **Unfav** | **Favour** | **Unfav** | **Favour** | **Unfav** |
| **Physician** | | | | | | | | |
| Favourable | 33 | 6 | 22 | 17 | 27 | 12 | 35 | 4 |
| Unfavourable | 1 | 37 | 8 | 30 | 6 | 32 | 5 | 33 |
| **Total** | 34 | 43 | 30 | 47 | 33 | 44 | 40 | 27 |
| Cohen's kappa | 0.82 | | 0.35 | | 0.53 | | 0.79 | |

### DISCUSSION

In this exploratory study, we have used a commonly used questionnaire for cancer patients, the EORTC QLQ-C30 (Aaronson et al, 1993). We cannot have any opinion about whether other instruments would have solved some of the problems in an easier way. However, we believe that the problems encountered, and the solutions suggested, are also relevant to other, presently available, questionnaires.

### Global health status/QoL

The traditional functional scales of the QLQ-C30, the global health status/QoL score and any of the symptom scales, could not effectively detect differences between treatment groups. The EORTC group recommends that the 'Global Health Status/QoL' scale is used as an overall summary measure (Fayers et al, 1995). This scale is capable of distinguishing between groups of patients assumed to differ in their overall QoL and to respond to changes in the health status (Bergman et al, 1991; Bjordal and Kaasa, 1992; Aaronson et al, 1993; Sigurdadóttir et al, 1996; Curran et al, 1997). Based upon the experiences of these 2 trials, this scale is, however, not sufficiently sensitive to provide relevant information about changes in several patients. The changes that were seen occurred more often in the question about 'overall physical condition' and not in the one about 'overall quality of life'. It is therefore unlikely that the modification made in later versions of QLQ-C30, replacing the question about 'overall physical condition' with 'overall health', will improve the ability to detect the changes (Fayers et al, 1995). With the criteria that the scores should change by more than one step (out of 12 steps) and exceed half a SD in order to be clinically relevant, many patients showed no change (even if a change appeared to be evident clinically and/or based upon the answers to the other questions of the questionnaire). There are different explanations for this apparent discrepancy, either that the 2 questions constituting the scale cannot identify the changes, or that no true changes occurred. Alternatively, the changes in performance and symptomatology we, as health professionals, register, and the patients themselves record in the remaining 28 questions, may not influence overall QoL. The positive and sometimes dramatic changes that may be seen in tumour-related symptoms together with improvements in several functions do not alleviate the severity of the disease and its ultimate outcome, with no comparative change in overall QoL, or, conversely, the patients' abilities to adequately cope with the negative changes that may accompany disease progression and/or that follows severe adverse effects may keep the overall QoL relatively stable. Patients may also shift their internal standards and values, or conceptualisation of perceived QoL, in addition to actual health state. This phenomenon, called response shift, is of fundamental importance to social and medical science (Sprangers and Schwartz, 1999). There is much evidence in the literature of paradoxical and counter-intrusive findings, which can be explained in terms of response shift. Patients with life-threatening diseases or disabilities report stable QoL (Andrykowski et al, 1993). Also, people with a severe chronic illness report the same levels of QoL as less severely ill patients and healthy persons (Cassileth et al, 1984; Breetvelt and Van Dam, 1991; Sprangers et al, 1999b). We have no method of identifying which of the above mentioned explanations prevail.

*© 2001 Cancer Research Campaign* 

### Alternative methods of analysing QLQ C-30

Rather than rely only on the patients' own perception of the 'Global Health Status/QoL' summary score, it appears important to use all, or at least most of, the information provided by the patients order to evaluate whether, and to what extent, the patient improves/continues to do well subsequent to an intervention. Alternative methods of scoring QoL data were thus tested. Each of the alternative methods successfully detected differences between treatment groups. It has been suggested that the individual patient should vary the weights that they attach to different aspects of life since QoL is highly individual (O'Boyle et al, 1992; Campell and Whyte, 1999). However, this approach is time and resource intensive and the effects of a treatment on a specific aspect can only be assessed for those patients choosing that area. In addition, the type and severity of symptoms caused by the disease and the treatments, the way patients react to their diagnosis, how they interact with relatives and friends and how their existential well-being is affected vary from individual to individual. Most of us accept that we cannot add up scores from different dimensions of a complex health profile (Cox et al, 1992). Yet, since in practice, improvements in some domains, but losses in others are seen in individual patients, we have to come up with practical solutions so that QoL measurements provide information that can facilitate the drawing of proper conclusions. We have in this paper described 3 ways of summarising the information in the questionnaire.

**QoL rating:** By using an older questionnaire, centred around problems such as pain, tiredness and distress in daily situations together with questions about existential well-being (Kaasa et al, 1988), we obtained good experience by allowing 2 raters to categorise a response based upon the scores at randomisation and during follow-up (Glimelius et al, 1989, 1994). Provided this rating is performed blindly as regards other information it provides an unbiased comparison of the outcome based upon the patients' own answers. In typical patients, when either a clear improvement, or a similarly clear deterioration, is seen in several aspects, this rating is very simple. In other instances, where the changes are small or particularly when they move in different directions, the rating may be difficult, and open for criticism in individual patients. When using the older instrument in patients with colorectal cancer, we found that the rating was easy in most patients (Glimelius et al, 1989, 1994). Using EORTC QLQ-C30, the rating was also straightforward in most patients with pancreatic and biliary cancer (Glimelius et al, 1996) but more often difficult in gastric cancer patients (Glimelius et al, 1997). This can, at least partially, be anticipated in the light of their differences in response to chemotherapy (Ross et al, 1997).

**Summary score:** Even if the blinded QoL ratings give an adequate, unbiased discrimination between the 2 groups, they are hampered by not being precisely defined and resource demanding. However, we do not find that the ratings are more uncertain than the objective response evaluation made by 2 independent radiologists on the bases of serial X-ray examinations (Labianca et al, 1996). More precise, and mathematically defined, criteria would have at least practical advantages. Although every type of recombination requires sets of weights (Cox et al, 1992; Matthews, 1993), the most simple and robust way is to make an unweighted sum of all scores. This is conceptually wrong, since all items are not equally important and all scales are not equally graded. The categorisation based upon the unweighted summation of all scores correlated well with the QoL rating, it showed a high agreement with the subjective response evaluation made by the physician, and it discriminated between the 2 groups. Therefore, it appears to be sufficiently reliable and valid to be used. Further analyses, however, illustrated that a weighting would provide more accurate estimates (data not shown). Some scales, like the role functioning scale (RF), appeared less relevant. These scales contained fewer levels (3 or 4 compared with 8 to > 10 for the others), thereby actually increasing their relative importance. In order to illustrate the relevance of weights, we arbitrarily reduced the relative influence of the RF scale by 50%. This simple manoeuvre reduced the problem substantially. It is likely that more accurate weights could be created, but we chose not to continue. The RF scale has been reformulated in later versions of the QLQ C-30 (Fayers et al, 1995). The inherent difficulties involved in the use of weights also prevented us from proceeding (Schipper, 1990; Cox et al, 1992; Matthews, 1993).

**Limited QoL categorisation:** 4 domains (physical functioning, emotional functioning, global health status/QoL and specific symptoms) were considered to be relatively more important in regard to potential differences between the treatment groups. An overall favourable outcome was analogous with the idea of a clinical benefit response (Rothenberg et al, 1996), considered to be present if the patient improved in at least one important aspect without deteriorating in others. The limited QoL categorisation could also successfully detect differences between treatment groups. It can be expected that the use of the limited QoL categorisation may result in more accurate detection of changes in QoL as it covers more aspects of patients' well being than the clinical benefit response rating. Furthermore, it is entirely defined by the patient and not a mixture of patient-defined and physician-defined endpoints. Liabilities of using the limited QoL categorisation are that it excludes some items that may be relevant for certain patients. Similar to the summary score, the limited QoL categorisation also is not conceptually adequate in explaining changes in QoL for individual patients, since no weights are utilised.

It can be concluded that the alternative methods of analysing the QLQ C-30 described here could discriminate between the 2 randomisation groups. A high agreement between the scales and with the subjective evaluation made by the physician were found. The agreements were also qualitatively the same in the 2 randomisation groups. We can not state whether one method is superior to any of the others, although it would appear as though the summary scores are less accurate than the others. It is, however, the simplest one to calculate. The patients' own summary, 'Global health status/QoL' was less discriminatory than either of the summary measures. More rigorous and empirically grounded procedures are needed for generating questionnaire summary scores and decision rules for classifying individual patients as QoL responders versus non-responders. The arbitrary scoring procedures employed here were then used for illustrative purposes only.

### Problems with compliance and attrition

Compliance with completing the questionnaire was in these studies no major problem, probably due to a long tradition to include QoL measurements in the trials and close monitoring by a research nurse. The difficulties to obtain completed questionnaires from patients with progressive disease and, thus, the poorest performance status, remain. If this drop-out can not be kept at a low level, it will disturb the analyses because of under-reporting of worsening QoL. Considerably lower levels of compliance have been reported in other studies (Fayers et al, 1997).

 *© 2001 Cancer Research Campaign*

The problems that can be seen when the attrition is not random are well illustrated in this study. Analyses of the average scores in interviewed patients will thus not reflect the outcome of the randomised population, but rather only those who make it to the next evaluation in a general condition good enough to complete the questionnaire.

This selective drop-out has been discussed by several researchers, and different analytic techniques have been suggested (Diggle and Kenward, 1994; Hopwood et al, 1994; Molenberghs et al, 1997; Moinpour et al, 2000). When reporting the results of the randomised trials, and used in this exploratory study, we chose to perform a categorisation of the patients into those with a favourable and those with an unfavourable QoL outcome. Non-interviewed patients were then all classified as non-responders. We did then not assign a particular QoL score to non-interviewed patients, and compared average scores for interviewed and non-interviewed patients, but rather based the categorisation upon the observation that non-interviewed patients were dead or terminally ill from the disease, i.e. they did not have a favourable outcome. A practical solution to the problem of this selective and thus potentially informative drop-out is to present data in different ways, and then discuss their relevance (Hopwood et al, 1994). For example, as can be seen in Table 2, the average scores during follow-up did not differ between the groups. When categorised, no differences were again seen if the proportion of responders was based only upon those interviewed (data not shown), whereas differences were seen if based upon all randomised patients (see Table 3). The conclusions are that chemotherapy with BSC is superior to BSC since more patients will have a favourable QoL outcome. They also live longer, and the average QoL during those extra months is, at least, not inferior to those experienced in the BSC group.

### Conclusions

The difficulties to obtain completed questionnaires in trials from patients with progressive disease will likely remain. A possible solution is to use a categorisation of patients into those with a favourable and those with an unfavourable QoL outcome and non-interviewed patients will be classified as non-responders. Although we can see advantages and disadvantages with each of the 3 alternative methods, they all appear to be useful for this purpose in patients with advanced cancers. For practical reasons, we do not advocate the QoL rating in large randomised trials. Although the limited QoL categorisation and summary score are not conceptually correct when interpreting individual patient's QoL, the use of these methods when assessing differences between treatment groups may prove to be beneficial. Research is warranted to replicate the findings as well as test similar methods of interpreting QoL in patients with advanced cancer at other sites.

### REFERENCES

Aaronson NK, Ahmedzai S, Bergman B, Bullinger M, Cull A, Duez NJ, Filiberti A, Flechtner H, Fleishman SB and Haes JCd (1993) The European Organization for Research and Treatment of Cancer QLQ-C30: A quality-of-life instrument for use in clinical trials in oncology. *J Natl Cancer Inst* **85**: 365–376

Andrykowski M, Brady M and Hung J (1993) Positive psychological adjustment in potential bone marrow transplant recipients: Cancer as a psychosocial transition. *Psycho-Oncol* **2**: 261–276

Bergman B, Sullivan M and Sörenson S (1991) Quality of life during chemotherapy for small cell lung cancer. I. An evaluation with generic health measures. *Acta Oncol* **30**: 947–957

Bernhard J, Cella D, Coates A, Fallowfield L, Ganz P, Moinpour C, Mosconi P, Osboa D, Simes J and Hürny C (1998) Missing quality of life data in cancer clinical trials: serious problems and challenges. *Stat Med* **17**: 517–532

Bernhard J, Hurny C, Maibach R, Herrmann R and Laffer U (1999) Quality of life as subjective experience: reframing of perception in patients with colon cancer undergoing radical resection with or without adjuvant chemotherapy. Swiss Group for Clinical Cancer Research (SAKK). *Ann Oncol* **10**: 775–782

Bjordal K and Kaasa S (1992) Psychometric validation of the EORTC core quality of life questionnaire, 30-item version and a diagnosis-specific module for head and neck cancer patients. *Acta Oncol* **31**: 311–321

Breetvelt I and Van Dam F (1991) Underreporting by cancer patients: The case of response shift. *Social Science & Medicine* **32**: 981–987

Campell S and Whyte F (1999) Quality of life in cancer patients participating in phase I clinical trials using SEIQoL-DW. Methodological issues. *Nursing Research* **30**: 335–343

Cassileth B, Lusk E and Tenaglia A (1984) A psychological comparison of patients with melanoma and other dermatological disordes. *Am Academy Dermatol* **7**: 742–746

Cella DF, Tulsky DS, Gray G, Sarafian B, Linn E, Bonomi A, Silberman M, Yellen SB, Winicour P, Brannon J, Eckberg K, Lloyd S, Purl S, Blendowski C, Goodman M, Barnicle M, Stewart I, McHale M, Bonomi P, Kaplan E, IV ST, CR Thomas J and Harris J (1993) The functional assessment of cancer therapy scale: Development and validation of the general measure. *J Clin Oncol* **11**: 570–579

Cox DR, Fitzpatrick R, Fletcher AE, Gore SM, Spiegelhalter DJ and Jones DR (1992) Quality-of-life assessment: Can we keep it simple? *J R Statist Soc* **155**: 353–393

Curran D, Fossa S, Aaronson N, Kiebert G, Keuppens E and Hall R (1997) Baseline quality of life of patients with advanced prostate cancer. *Eur J Cancer* **33**: 1809–1814

de Haes JC, van Knippenberg FC and Neijt J (1990) Measuring psychological and physical distress in cancer patients: Structure and application of the Rotterdam Symptom Checklist. *Br J Cancer* **62**: 1034–1038

Detmar SB, Aaronson NK, Wever LD, Muller M and Schornagel JH (2000) How are you feeling? Who wants to know? Patients' and oncologists' preferences for discussing health-related quality-of-life issues. *J Clin Oncol* **18**: 3295–3301

Diggle P and Kenward MG (1994) Informative drop-out in longitudinal data analysis. *Appl Statist* **43**: 49–93

Fayers P, Aaronson N, Bjordal K and M Sullivan (eds) (1995) EORTC QLQ-C30 Scoring Manual. EORTC Quality of Life Study Group: 1–25

Fayers PM, Hopwood P, Harvey A, Girling DJ, Machin D and Stephens R (1997) On behalf of the MRC Cancer Trials Office. Quality of life assessment in clinical trials–Guidelines and a checklist for protocol writers: the U.K. Medical Research Council Experience. *Eur J Cancer* **33**: 20–28

Glimelius B, Hoffman K, Olafsdottir M, Påhlman L, Sjödén P and Wennberg A (1989) Quality of life during cytostatic therapy for advanced symptomatic colorectal carcinoma. A randomized comparison of two regimens. *Eur J Cancer Clin Oncol* **25**: 829–835

Glimelius B, Graf W, Hoffman K, Påhlman L, Sjödén PO and Wennberg A (1992) General condition of asymptomatic patients with advanced colorectal cancer receiving palliative chemotherapy: A longitudinal study. *Acta Oncol* **31**: 645–651

Glimelius B, Hoffman K, Graf W, Påhlman L, Sjödén P-O and Wennberg A (1994) Quality of life during chemotherapy in symptomatic patients with advanced colorectal cancer *Cancer* **73**: 556–562

Glimelius B, Hoffman K, Sjödén P-O, Jacobsson G, Sellström H, Enander L-K, Linné T and Svensson C (1996) Chemotherapy improves survival and quality of life in advanced pancreatic and biliary cancer. *Ann Oncol* **7**: 593–600

Glimelius B, Ekström K, Hoffman K, Graf W, Sjödén P-O, Haglund U, Svensson C, Enander L-K, Linné T, Sellström H and Heuman R (1997) Randomized comparison between chemotherapy plus best supportive care with best supportive care in advanced gastric cancer. *Ann Oncol* **8**: 163–168

Guyatt GH, Veldhuyzen Van Zanten SJ, Feeny DH and Patrick DL (1989) Measuring quality of life in clinical trials: a taxonomy and review. *CMAJ* **140**: 1441–1448

Gunnars B, Nygren P and Glimelius B (2001) Assessment of quality of life during chemotherapy. *Acta Oncol* **40**: 175–184

Hopwood P, Stephens RJ and Machin D (1994) Approaches to the analysis of quality of life data: experiences gained from a Medical Research Council Lung Cancer Working Party palliative chemotherapy trial. *Qual Life Res* **3**: 339–352

Juniper EF, Guyatt GH, Willan A and Griffith LE (1994) Determining a minimal important change in a disease-specific Quality of Life Questionnaire. *J Clin Epidemiol* **47**: 81–87

*© 2001 Cancer Research Campaign*

Kaasa S, Mastekaasa A, Stokke I and Naess S (1988) Validation of a quality of life questionnaire for use in clinical trials for treatment of patients with inoperable lung cancer. *Eur J Clin Oncol* **24**: 691–701

Labianca R, Pancera G and Luporini G (1996) Factors influencing response rates for advanced gastrointestinal cancer chemotherapy. *Ann Oncol* **7**: 901–906

Lydick E and Epstein RS (1993) Interpretation of quality of life changes. *Qual Life Res* **2**: 221–226

Matthews JNS (1993) A refinement to the analysis of serial data using summary measures. *Stat Med* **12**: 27–37

Moinpour CM, Sawyers Triplett J, McKnight B, Lovato LC, Upchurch C, Leichman CG, Muggia FM, Tanaka L, James WA, Lennard M and Meyskens Jr. FL (2000) Challenges posed by non-random missing quality of life data in an advanced-stage colorectal cancer clinical trial. *Psychooncology* **9**: 340–354

Molenberghs G, Kenward MG and Lesaffre E (1997) The analysis of longitudinal ordinal data with nonrandom drop-out. *Biometrika* **84**: 33–44

Morris J, Perez D and McNoe B (1998) The use of quality of life data in clinical practice. *Qual Life Res* **7**: 85–91

Nordic Gastrointestinal Tumor Adjuvant Therapy Group (1993) Biochemical modulation of 5-fluorouracil: A randomized comparison of sequential methotrexate, 5-fluorouracil and leucovorin versus sequential 5-fluorouracil and leucovorin in patients with advanced symptomatic colorectal cancer. *Ann Oncol* **4**: 235–241

O'Boyle CA, McGee H, Hickey A, O'Malley K and Joyce CR (1992) Individual quality of life in patients undergoing hip replacement. *Lancet* **339**: 1088–1091

Osboa D, Rodrigues G, Myles J, Zee B and Pater J (1998) Interpreting the significance of changes in health-related quality-of-life scores. *J Clin Oncol* **16**: 139–144

Ross PJ, Webb A, Cunningham D, Prediville J, Norman AR and Oates J (1997) Infusional 5-fluorouracil in the treatment of gastrointestinal cancers: The Royal Marsden Hospital experience. *Ann Oncol* **8**: 111–115

Rothenberg aL, Moore MJ, Cripps MC, Andersen JS, Portenoy RK, Burris HA, Green MR, Tarassoff PG, Brown TD, Casper ES, Storniolo AM and Hoff DDV (1996) A phase II trial of gemcitabine in patients with 5-FU-refractory pancreas cancer. *Ann Oncol* **7**: 347–353

Sanders C, Egger M, Donovan J, Tallon D and Frankel S (1998) Reporting on quality of life in randomised controlled trials: bibliographic study. *BMJ* **317**: 1191–1194

Schag CAC, Ganz PA and Heinrich RL (1991) Cancer Rehabilitation Evaluation System-Short Form (CARES-SF): A cancer specific rehabilitation and quality of life instrument. *Cancer* **68**: 1406–1413

Schipper H (1990) Quality of Life: Principles of the clinical paradigm. *J Psychosocial Oncol* **8**: 171–185

Sigurdadóttir V, Bolund C and Sullivan M (1996) Quality of life evaluation by the EORTC questionnaire technique in patients with generalized malignant melanoma on chemotherapy. *Acta Oncol* **35**: 149–159

Sprangers MA and Schwartz CE (1999) Integrating response shift into health-related quality of life research: a theoretical model. *Soc Sci Med* **48**: 1507–1515

Sprangers M, te Velde A and Aaronson N (1999a) The construction and testing of the EORTC colorectal cancer-specific quality of life questionnaire module (QLQ-CR38). *Eur J Cancer* **35**: 238–247

Sprangers M, Van Dam F, Broersn J, Lodder L, Wever L, Visser M, Oosterveld P and Smets E (1999b) Revealing response shift in longitudinal research on fatigue-the use of the thentest approach. *Acta oncol* **38**: 709–718

Troxel A, Fairclough D, Curran D and Hahn E (1998) Statistical analysis of quality of life with missing data in cancer clinical trials. *Stat Med* **17**: 653–666

Wilke H, Preusser P, Stahl M, Harstrick A, Meyer HJ, Achterrath W, Schmoll HJ and Seeber S (1991) Etoposide, folinic acid, and 5-fluorouracil in carboplatin-pretreated patients with advanced gastric cancer. *Cancer Chemother Pharmacol* **29**: 83–84

*© 2001 Cancer Research Campaign*
